# Dutch orthopedic thromboprophylaxis: a 5-year follow-up survey

**DOI:** 10.1080/17453670902807441

**Published:** 2009-02-01

**Authors:** Harmen B Ettema, Marieke C Mulder, Michael T Nurmohamed, Harry R Büller, Cees C P M Verheyen

**Affiliations:** ^1^Department of Orthopedic Surgery and Traumatology, Isala ClinicsZwollethe Netherlands; ^2^Department of Internal Medicine, VU University Medical CenterAmsterdamthe Netherlands; ^3^Department of Vascular Medicine, Academic Medical Center, University of AmsterdamAmsterdamthe Netherlands

## Abstract

**Background and purpose** Previous surveys in the Netherlands have revealed that guidelines regarding orthopedic thromboprophylaxis were not followed and that a wide variation in protocols exists. This survey was performed to assess the current use of thromboprophylactic modalities and to compare it with the results of a previous survey.

**Methods** All departments of orthopedic surgery in the Netherlands were sent a follow-up survey on venous thromboprophylaxis, and the data obtained were compared to the results of a survey performed 5 years earlier.

**Results** All departments used pharmacological thromboprophylaxis following arthroplasties of the hip and knee. Low-molecular-weight heparin (LMWH) was used most frequently (79%) of the departments, followed by fondaparinux (13%). 5 years earlier, coumarin treatment was the predominant prophylaxis (79%). All departments prescribed pharmacological prophylaxis after femoral and tibial fractures; 78% used LMWH. Prophylaxis was continued for 6 weeks in 85% of cases. LMWH treatment was initiated on the day before surgery in 31% of cases (65% in the previous survey), perioperatively in 55%, and in the evening following surgery in 24%. In general, for daycare surgery and arthroscopies either no prophylaxis was given or a LMWH was given for 1 day. After anterior cruciate ligament reconstruction, 94% of departments prescribed some form of pharmacological prophylaxis.

**Interpretation** The use of pharmacological prophylaxis after arthroplasty of the hip and knee and also after fracture surgery around the hip and knee is common practice in the Netherlands. In 5 years, the widely used coumarin derivates have been largely replaced with LMWH.

## Introduction

Deep vein thrombosis (DVT) and pulmonary embolism (PE) are common complications after orthopedic surgery, especially after arthroplasties and fracture surgery (Geerts et al. 2004). Dutch national guidelines (Büller et al. 2000) have not been followed (Schonenberg et al. 2003, Ettema et al. 2005).

Since more attention has been drawn to thromboprophylaxis and new pharmacological modalities have been introduced, we conducted this survey to assess the current situation. We also compared it with the results of a survey from 5 years earlier (Ettema et al. 2005). As in that study, we concentrated on hip and knee arthroplasties. Additional questions were asked regarding daycare, short stay, fractures, and plaster cast immobilization.

## Methods

A questionnaire on orthopedic departmental protocols for perioperative thromboprophylaxis was sent to all Dutch orthopedic departments. It was tailored to fit a similar one performed in 2002 (Ettema et al. 2005). Practice profile, and current choice, initiation, and duration of thromboprophylaxis after several orthopedic procedures were assessed.

In 2007, a package with the questionnaire, a cover letter, and a stamped addressed envelope were sent to all Dutch orthopedic departments. Non-respondents were sent a reminder after 4 months, and were contacted by telephone if necessary. Categorical data and dichotomous variables were summarized as percentages of the responding departments. For key features, the results were compared with those in the 2002 database, which had a response rate of 84 out of 110 departments (79%).

## Results

81 of 96 departments answered the questionnaire properly. All respondents stated that they had a specific departmental protocol on thromboprophylaxis.

### Hip and knee arthroplasty

All departments used pharmacological thromboprophylaxis during hospital admission after hip and knee arthroplasties (Table 1). Low–molecular-weight heparin (LMWH) monotherapy had replaced vitamin K antagonist (VKA) as the predominant agent used by the responding departments in 2007 after elective hip and knee arthroplasty.

**Table 1.** Thromboprophylactic regimens used after elective arthroplasties (%)

	In hospital only		In hospital and extended thromboprophylaxis							
Arthroplasty	LMWH		LMWH		LMWH and VKA's **^a^**		fondaparinux		Aspirin	
	2002	2007	2002	2007	2002	2007	2002	2007	2002	2007
Total hip	2	None	17	79	80	7	NA	14	1	0
Revision hip	2	None	16	80	81	7	NA	13	1	0
Total knee	2	None	20	79	77	8	NA	14	1	0
Revision knee	2	None	18	80	79	7	NA	13	1	0
Hemi knee	9	3	8	78	82	7	NA	12	1	0
Average	3	1	17	79	79	7	NA	13	1	0

**^a^** LMWH until adequate INR was reached; in 2002, 12% of departments used a coumarin only.

In all departments, coumarines (when prescribed) were combined with a LMWH during the first days of treatment until an adequate internationalized normalized ratio (INR) was reached. The synthetic Xa inhibitor fondaparinux was given in 13% of hip and knee arthroplasties. Since fondaparinux was not available during the survey 5 years earlier, no comparison can be made. None of the respondents used aspirin. LMWH prophylaxis was given after discharge for 3–4 weeks in 9% of patients/departments, 6 weeks in 85%, and 2–3 months in 6%. Coumarines were continued for 6 weeks in 60% of patients/departments and 3 months in 40%. 5 years earlier, coumarins had been continued for 3 months in 65% of cases/departments. In the present study, fondaparinux was prescribed for 3–4 weeks in 55% of patients/departments and 5–6 weeks in 45%.

### Fractures of the proximal femur and tibia

All departments used pharmacological prophylaxis after osteosynthesis or arthroplasty of hip and proximal femur fractures, and after internal fixation of proximal tibia fractures (Table 2). In 2007, as with elective arthroplasties, LMWH had come to replace coumarins as the mainstay of thromboprophylaxis after hemiarthroplasty of the hip (compared to 5 years earlier). Prophylaxis was continued for 4–6 weeks in 88% of patients/departments and for 2–3 months in 9%. In 2007 thromboprophylaxis was not continued after discharge in 3% of the departments, which was less than in 2002 (9%).

**Table 2.** Thromboprophylactic regimens used after fracture surgery. Values are percentages

	In hospital only		In hospital and extended out-of-hospital thromboprophylaxis					
			LMWH		LMWH and VKA's **^a^**		fondaparinux	
Trauma procedure	2002	2007	2002	2007	2002	2007	2002	2007
Hemi hip arthroplasty	9	3	15	78	75	9		10
Hip, internal fixation		1		81		9		9
Proximal femur, internal fixation		4		78		8		10
Proximal tibia, internal fixation		12		79		8		5

**^a^** LMWH until adequate INR was reached.

### Initiation of treatment

LMWH treatment was started on the evening before surgery in 31% of patients/departments, 2 h preoperatively in 23%, less than 6 h postoperatively in 22%, and in the evening following surgery (regardless of the time of surgery) in 24% of patients/departments. In 2002, 65% of all LMWH therapy was started on the evening before surgery and in 21% of cases/departments it was started 2 h preoperatively. Coumarins were started on the day of surgery in 46% of patients/departments and later in 54%. Fondaparinux was always initiated 6–12 hours postoperatively (as recommended by the manufacturer).

### Day care, arthroscopy, and anterior cruciate ligament (ACL) reconstruction

When treating patients in daycare surgery (including arthroscopy of the knee), compared to 5 years earlier an increased number of departments did not use any prophylaxis in 2007 (Table 3). After ACL reconstruction 50% of departments used a LMWH for 1–3 days (or the period of admission), 5% for 1 or 2 weeks, 2% for 3–4 weeks, and 35% of departments used LMWH prophylaxis for 6 weeks. 1 hospital prescribed fondaparinux for 2 weeks. Another hospital used a coumarin for 6 weeks. No form of prophylaxis was given in 6% of cases/departments.

**Table 3.** Thromboprophylactic regimens used after daycare surgery and knee arthroscopy (%)

	No prophylaxis		LMWH once or during admission		Other	
Procedure	2002	2007	2002	2007	2002	2007
Day-care	40	64	59	34 **^a^**	0	2 **^c^**
Arthroscopy	40	4	59	49 **^b^**	1	3 **^d^**

**^a^** 54% initiation preoperatively,

**^b^** 52% initiation preoperatively,

**^c^** 1 department used fondaparinux for 2 weeks and 1 LMWH for 5 days,

**^d^** 1 department used LMWH for 5 days, 1 department recommended 6 weeks of vitamin K antagnist as prophylaxis and 1 department recommended 14 days of fondaparinux.

### Plaster cast immobilization (Table 4)

During lower leg immobilization, some form of pharmacological prophylaxis was given in 70% of cases/departments. During immobilization of both knee and ankle, thromboprophylaxis was used in 94% of the departments. In 2002, most departments prescribed a coumarin derivate but by 2007 this had generally been replaced with a LMWH.

**Table 4.** Thromboprophylactic regimens used with immobilization of the lower extremity (%)

	No prophylaxis		LMWH		LMWH without weight bearing		fondaparinux		coumarin	
Type of immobiliszation	2002	2007	2002	2007	2002	2007	2002	2007	2002	2007
Below the knee only	50	30	10	52	3	13	NA	4	37	1
Above the knee and ankle	11	6	16	84	5	5	NA	3	68	2

### NSAIDs, aspirin and coumarins

83% of the departments discontinued aspirin for 3–10 days before surgery. Continued use of aspirin until the day of surgery was a reason for delayed surgery in 26% of these departments. NSAIDs were discontinued for 2–10 days before surgery in only 40% of the hospitals; non-compliance with this protocol was a reason for cancellation of surgery in only 10% of the departments. Coumarin therapy was interrupted in all departments for 2–10 days. Patients were managed with vitamin K or clotting factor infusions in 58% of departments, or surgery was delayed when it was not discontinued (42% of departments).

### Mechanical prophylaxis

In addition to pharmacological prophylaxis, some hospitals used mechanical prophylactic devices. Elastic stockings were used after total hip arthroplasty in 8% of departments as compared to 20% in 2002, and after knee arthroplasty in 5% of departments as compared to 11% previously. Intermittent pneumatic compression was not employed in any of the departments that responded to the questionnaire.

Of all the departments, 93% did not prescribe any standard prophylaxis for patients below 16 years of age, 5% treated these patients as adults, and 2% had a more complicated protocol.

The most frequently prescribed LMWH was nadroparin (76%), followed by dalteparin (19%) and enoxaparin (5%) in their standard prophylactic doses (98%). This has not changed appreciably in 5 years.

## Discussion

Our follow-up survey reveals that within 5 years, vitamin K antagonists have been largely replaced with LMWH and fondaparinux for the prevention of venous thromboembolic events (VTEs) in orthopedic surgery in the Netherlands. There is still considerable variation in protocols among different departments. The Dutch consensus document “Deep venous thrombosis and pulmonary embolism” recommends thromboprophylaxis with a LMWH for a period of 6 weeks after major orthopedic surgery of the hip and knee, and VKA is considered an alternative only (Büller et al. 2000). Generally, a LMWH is prescribed after arthroplasty of the hip and knee; this shows that compliance with the consensus is better than in the previous two surveys (Schonenberg et al. 2003, Ettema et al. 2005). Since the consensus originated in 2000, it does not include new anticoagulants such as fondaparinux. Newer guidelines (Geerts et al. 2004) do favor fondaparinux after arthroplasty and hip fracture surgery. Clearly, a considerable number of orthopedic departments have looked ahead and introduced these synthetic agents before any new national guidelines appear. We believe these results are representative because of the high response rate.

We found that LMWH is generally started perioperatively (between 2 h before and 6 h after surgery) in the Netherlands. The remaining departments start either on the day before or the evening after surgery. It appears that a preoperative start is no more effective than a postoperative start (Strebel et al. 2002). A perioperative start is apparently more effective, but this is counterbalanced by a marked increase in the risk of major bleeding in comparison with a preoperative or postoperative regimen (Strebel et al. 2002). Although international guidelines advise against the use of spinal and epidural blocks in patients on LMWH perioperatively (Horlocker et al. 2003), the Dutch practice does not contradict the Dutch guidelines for single-shot spinal anesthesia (De Lange et al. 2004).

Extended pharmacological thromboprophylaxis is standard after hip and knee arthroplasty and hip fracture surgery. A meta-analysis showed a favorable effect of LMWH with a reduction in total thrombosis after hip arthroplasties (asymptomatic as well as symptomatic) when compared to placebo or no prophylaxis after 30–42 days of treatment. The data regarding knee arthroplasty are less obvious (Eikelboom et al. 2001).

Although favorable results have been reported in a number of studies with mechanical methods of prophylaxis (Kaempffe et al. 1991, Francis et al. 1992), pneumatic compression devices were no longer used by the responders in our study. Elastic stockings are used, but only as an adjuvant to a pharmacological regimen.

The use of prophylaxis after smaller procedures such as arthroscopy of the knee or ACL reconstruction remains controversial (Nurmohamed 2007, Verheyen 2007); the risk after arthroscopy of the knee appears to be low (Ettema et al. 2006, Hoppener et al. 2006) whereas the risk of thrombosis after ACL reconstruction is unknown. This uncertainty is reflected in the wide variation in regimens following these procedures in the responding departments. Much of the same applies to plaster cast immobilization: the national guidelines recommend the use of prophylaxis after above–the-knee casts, but not with below-knee casting. Furthermore, the ACPP consensus statement does not recommend routine prophylaxis with below-knee fractures (Geerts et al. 2004). Even so, a recent meta-analysis of randomized controlled trials investigating thromboprophylaxis with plaster cast immobilization has shown a favorable effect of LMWH on asymptomatic endpoints (Ettema et al. 2008).

In summary, the use of pharmacological prophylaxis after arthroplasty of the hip and knee and also after fracture surgery around the hip and knee is common practice in the Netherlands. Generally speaking, it is continued after discharge. LMWH or fondaparinux is mainly used, whereas 5 years ago coumarin derivates were the preferred treatment. There still remains a wide variability in regimens after smaller procedures such as arthroscopy and ACL reconstruction and after immobilization of the leg.
